# Methods to improve quality performance at scale in lower- and middle-income countries

**DOI:** 10.7189/jogh.08.021002

**Published:** 2018-12

**Authors:** György Fritsche, John Peabody

**Affiliations:** 1 The World Bank Group, Washington, D.C., USA; 2 QURE Health Care, San Francisco, California, USA

## Abstract

Universal Health Coverage is one of the Sustainable Development Goal targets. But coverage without quality health services limits benefits to populations. Performance-based financing programs (PBF) use strategic purchasing of services to expand coverage and promote quality by measuring quality and rewarding good performance. The widespread presence of PBF programs in lower and middle-income countries provide an opportunity to introduce and test new approaches for measuring and improving quality at scale. This article describes four approaches to improve quality of health services at scale in PBF programs. These approaches looked at structural and process measures of quality as well as outcome measures like patient satisfaction. Three types of tools were used in these approaches: clinical vignettes, competency tests and patient satisfaction surveys. Specific tools within each of the approaches are used in Kyrgyzstan, Cambodia, Democratic Republic of Congo and the Republic of Congo.

In September 2015, the United Nations General Assembly adopted the Sustainable Development Goals. Goal 3, sub goal 3.8 is achieving Universal Health Coverage (UHC) [1]. The path to UHC involves securing resources, reducing the reliance on direct out of pocket payments for health services, and improving efficiency and equity [2]. Moving towards UHC involves defining a benefit package, financing this benefit package in such a way that the poorest can also access these services [3], and applying strategic purchasing to ensure that this benefit package is delivered.

However, low quality of services yield lesser health benefits and therefore are less effective [4]. The World Health Organization estimates that between 20-40% of all health spending is wasted due to inefficiencies and poor quality [2]. This means that even among the most successful UHC programs, poor quality of services negates the gains from expanded coverage and benefits. In contrast, higher quality of care has a particularly large effect on under-five mortality, especially in poor countries [5].

Quality, it is recognized, is critically important for the post-Millennium Development Goal (MDG) agenda [6]. Traditional approaches such as provider training and supervision have at best a limited impact, requiring a better or different way of doing things [4,7-9]. Hence the focus on effective and quality coverage and benefits.

The widespread presence of PBF programs in lower- and middle-income countries (LMIC), an increasing number at scale in which quantity and quality are purchased from autonomous health facilities, offer a window of opportunity to leverage not only quantity but also quality of services delivered.

This article is organized as follows: first, we will provide a background by describing quality of care as a concept, then we will discuss PBF, followed by the experience measuring quality in PBF programs. Thereafter, we will describe a theory of change underlying the use of quality measures in the context of PBF, after which we will describe the novel quality measurement methods. Finally, we will provide four country examples of the application of these tools. In the discussion section, we will reflect on some challenges and possible caveats.

## Quality of care

Quality of health services is variable everywhere, but is especially low in lower- and middle-income countries [8,10-15]. Through the World Bank’s Service Delivery Indicator (SDI) Surveys carried out in various Sub-Saharan African countries, the poor state of delivering quality health services is apparent (**Table 1**).

**Table 1:** Service Delivery Indicator (SDI) comparator table http://www.sdindicators.org/

	Niger (2015)	SDI Avg.	Madagascar (2016)		Mozambique (2015)	Tanzania (2014)	Nigeria (2013)	Togo (2013)	Uganda (2013)	Kenya (2013)	Senegal (2010)
Caseload (per provider per day)	9.8	**8.8**		5.2	17.4	7.3	5.2	5.2	6.0	15.2	-
Absence from facility (% providers)	33.1	**28.6**		27.4	23.9	14.3	31.7	37.6	46.7	27.5	20
Diagnostic accuracy (% clinical cases)	31.5	**50.1**		30	58.3	60.2	39.6	48.5	58.1	72.2	34
Adherence to clinical guidelines (% clinical guidelines)	17.5	**35.9**		31	37.4	43.8	31.9	35.6	41.4	43.7	22
Management of maternal and neonatal complications (% clinical guidelines)	12.0	**27.4**		21.9	29.9	30.4	19.8	26.0	19.3	44.6	-
Drug availability (% drugs)	50.4	**54.4**		48	42.7	60.3	49.2	49.2	47.2	54.2	78
Equipment availability (% facilities)	35.9	**61.3**		62	79.5	83.5	21.7	92.6	21.9	76.4	53
Infrastructure Availability (% facilities)	13.3	**40.6**		28.4	34	50	23.8	39.2	63.5	46.8	39

An early established global framework for understanding quality of health services is that of Donabedian in which a distinction is made between structural, process and outcome measures of quality [16]. Structural measures are the inputs necessary for providing quality services such as equipment, drugs and trained providers; process measures are the actions and activities of health providers, and outcomes are the results of medical action such as patient satisfaction, improved health, disability or death [16]. Process measures of quality, ie, what happens between the patient and the provider also referred to as provider effort are commonly associated more closely with health outcomes [8,9,17]. Frequently, structural and process measures of quality are present conjointly to lead to desired outcomes [18-20]. As an example, in the case of treating pulmonary tuberculosis: tuberculosis drug availability (structural element) is closely linked to making the right diagnosis, the right explanations to the patient, and the delivery of effective direct observed treatment (process elements) and should all be present to yield the highest possible chance for successful cure (outcome).

The strength of this framework, for example in the case of moderately severe diarrhea in a young child, is that appropriate care can be effectively “provided under a palm tree” by a “knowledgeable person” (structural) if that provider makes the right diagnosis, and provides correct advice using integrated management of childhood illnesses (process). A unilateral focus on structural quality measures leads to measuring the wrong things [8,17,21]. Instead, we will consider the systemic nature of health systems and by extension health service delivery when considering interventions that influence the delivery of quality service. PBF is an illustration of a systemic intervention designed to work towards Universal Health Coverage, not merely by addressing volume, but influencing the structures of quality health service delivery [22-26].

## Performance-based financing and strategic purchasing

PBF is a health financing approach that originated in Cambodia in the late 1990s [27-32] and evolved to its current form through various iterations in countries like Democratic Republic of the Congo (DRC), Rwanda and Burundi [24]. In Rwanda, after a series of successful PBF pilot programs [33-37], PBF was scaled up through a mix of government and development partner financing and operational efforts. A rigorous impact evaluation [38] showed significant results on volume and quality of maternal and child health services [39] and HIV services [40]. The PBF approach which evolved between 2005-2009 in Rwanda was further developed in Burundi [41-44] and other countries such as DRC [45], Cameroon and Nigeria [24].

Key features of these PBF approaches are (a) defining basic and complementary health packages for strategic purchasing; (b) strategic purchasing adjusted through measures of service quality; (c) purchasing from public, private and quasi-public providers; (d) involvement of national and sub-national health administration in stewardship, supervision, quality measurements and other technical and managerial supportive tasks; (e) community client satisfaction surveys to strengthen community voice; (f) rigorous internal and external verification mechanisms; (g) creation of fiscal space through linking to national health financing strategies, since 2015 also assisted through the Global Financing Facility [46].

PBF approaches are systemic approaches [22], and can best be conceptualized as a constellation of leveraging, health system strengthening and accountability mechanisms. These approaches work best when embedded in overall health reforms in which various system pillars are simultaneously addressed such as human resources, health information systems, the pharmaceutical sector and fiscal space for health (**Table 2**) [23,25,26,47,48].

**Table 2:** Hierarchy of system interventions in performance-based financing programs (PBF) approaches driving results*

Health system level	Intervention	Intended result
Central	Central technical support unit	Strengthened stewardship
	Defining benefit packages; defining quality measures; costing out intervention; setting subsidies	
	Managing ICT	
	Strategic purchasing	
	Securing budgets	
	Technical support for purchasing agencies	
	Contract counter-verification agent	
	Coordination	
Sub-national	Formative supervision	Strengthened governance
	Application of quantified quality checklists including knowledge and competency tools (each quarter)	Ownership of results
	Capacity building	Strengthened administrative accountability
	Coordination	
Health facility	Decentralized facility financing (based on volume and quality of services)	Enhanced supply of quality essential health services
	Business planning	Increased financial accessibility for essential health services
	Delivery of essential health packages at health; center/community and hospitals	Fee exemptions for indigents
	**Focus on quality** (structural; process and outcome)	**Enhanced structural quality**
		**Enhanced knowledge and competency of providers**
Community	Community client satisfaction surveys	Strengthened community voice
	Participation in community health committees	Community ownership
	Community health workers organized for outreach activities	Enhanced governance

*Within this hierarchy of systemic interventions of PBF approaches, the bold faced elements are targeted through innovative methods to enhance quality – the topic of this paper.

**Figure 1:**
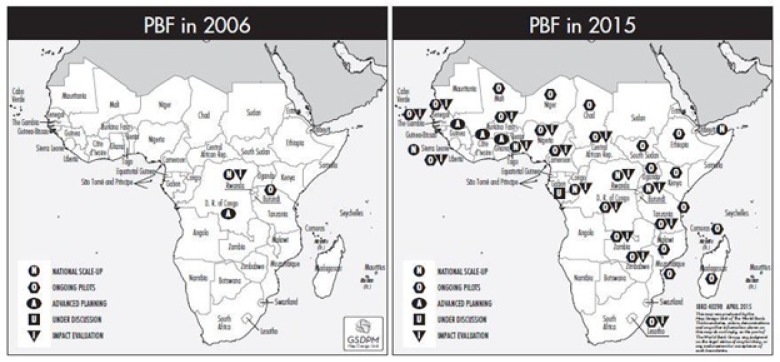
Rapid expansion of performance-based financing programs (PBF) in SSA 2006-2015.

PBF approaches have expanded and reached scale in many countries (eg, in Sub-Saharan Africa (SSA), Central and South-East Asia and the Caribbean) making it possible to look at the impact of strategic purchasing for UHC and any novel or promising quality interventions. The map below (**Figure 1**) shows the rapid expansion of PBF approaches in SSA. Some programs such as the Nigerian State Health Investment Project or the Health System Development Project in DRC, for example, cover significant populations; 33 million and 22 million respectively. Cameroon is slated to scale up PBF nationwide during 2019 to cover a population of 24 million. A project in Kyrgyzstan covers all 63 Rayon (district) hospitals, and a project in Cambodia, a country with 16 million, will during 2018 cover all public health centers and hospitals, including the sub-national health administration.

## PBF and quality of care

Quality adjusted strategic purchasing of essential health services is an essential aspect of PBF approaches. Since the start, PBF approaches have included the use of a quantified quality checklist applied quarterly to each contracted health facility [24]. Such checklists differ depending on level (health center/community vs hospital) and context, and typically involve over 120 data elements [21]. They have proved popular both because it is easier to administer and verify data related to infrastructure and supplies and as the initial startup funds are often tied to making improvements in these structural elements.

Indeed, health facilities in poor countries, due to chronic underfunding, lack many structural quality features that are routine for health facilities in countries with more resources. For instance, if a service availability and readiness assessment in DRC shows that only 13% of public and private health facilities have magnesium sulphate (an essential drug for managing morbidity and mortality due to pregnancy related eclampsia) or it shows that less than 12% of health facilities can provide basic emergency obstetric care, we clearly have a problem [49]. However, this problem becomes more complex when next to service availability and readiness we consider provider knowledge and competency. In SSA these are measured through SDI surveys, which are done in SSA (**Table 1**). In addition to significant gaps in structural quality (as an extreme example, 46.7% of providers are absent in Uganda), such surveys show a huge variation in provider knowledge and competency. For instance, on average in nine SSA countries only 27.4% of providers adhere to guidelines for neonatal and maternal health complications, 35.9% adhere to clinical guidelines for diarrhea, pneumonia and malaria while diagnostic accuracy for these conditions was only 50%. Studies in India show a similarly dire picture [8,13,15]. Here is a general recognition that most checklists are focused upon structural factors at the expense of process measures and accordingly, the various quality checklists have increased weights for process measures of quality. In Nigeria, for example, process elements form 30% of the weight (103 out of 345 points available) at health center level and 41% of the weight at hospital level (240 out of 587 points available) of the overall quality checklist. These process measures are mostly based on file or record reviews and sometimes on observations. They are known to be difficult to ascertain or to counter-verify. For instance, in maternal medicine, a commonly used process measure, “well-filled partogram”, is frequently filled after delivery when missing partogram elements can be filled by knowledgeable providers before a verification would take place [50]. In a similar vein, criterion-based medical audits - although perhaps working in different contexts [51] - also break down when used in PBF approaches due to a similar propensity for gaming the right answer instead of recording the actual practice.

## Integrating further quality measures into PBF

Below, we reflect on a selection within the armamentarium of methods to measure and document quality processes and outcomes and options to integrate these methods in such a manner that they are effective along three dimensions: (a) efficient; can be added to an existing PBF program at minor incremental cost; (b) responsive; able to document an improvement or lead to an advancement on the previous state of affairs, and (c) scalable; can be done at the population level.

The operationalization of these schemes needs to be undertaken in such a way that the schemes can really strengthen indices for health care quality and work in the environment of a PBF operation, and validated by regular and rigorous assessments (backed by third-party verifications), performance feedback, elements of supportive supervision and public benchmarking. We postulate that PBF environments offer a fertile context for behavior change as they mix various behavioral triggers to practice improvement [8,14,52,53].

We looked at five methods used for assessing medical process quality: (1) chart review, (2) vignettes, (3) direct observations, (4) mystery patients, and (5) exit interviews [8]. Chart reviews abstract practice documentation from the patient’s chart using explicit evidence based criteria. Vignettes are standardized medical cases, where providers care for the same patients to facilitate comparison of practices; they are done in various ways from full cases akin to role-play, or as a short answer, effecting a written exam. Mystery patients are trained actors who present unannounced with certain symptoms and are trained to observe and report on medical actions performed on them. Finally, patients can be interviewed on what has been done to them when leaving a consultation room. These methods are variably used for research and for audits, for accreditation or teaching. Considering a pre-existing PBF program, efficiency, responsiveness and scalability, the following options to measure process and outcome quality are available in **Table 3**.

**Table 3:** Comparison of options for measuring process or outcome quality in a performance-based financing programs (PBF) program

Method	Advantage	Disadvantage	Scope (efficiency; responsiveness and scalability)
Chart review	Readily available; currently practiced	Documentation is highly variable; Gaming is easy and Case mix is uncontrolled	Inefficient but responsive and scalable
Vignettes	Cases are standardized for benchmarking; inexpensive and readily scalable; can also be used for rare conditions	Limited experience in a PBF environment; concerns of ‘know-do’ gap; vignettes are a generic term and all vignettes are not the same	Efficient, responsive and scalable; linked to better outcomes
Direct observation	Assesses competency	Limited experience in a PBF environment, difficult to scale	Not efficient, however could be made responsive to key conditions, Difficult to scale
Mystery patient	Avoiding Hawthorne effect (‘Gold Standard’)	No experience in a PBF environment; limited range of conditions can be simulated; training and inter-rater reliability a challenge	Not efficient, difficult to make responsive, difficult to scale
Exit interview	Patient perspective on the care provided can be quantified providing information on effort	No experience in a PBF environment	Theoretically possible, but probably not practical due to PBF context. Doubtful efficient, doubtful responsive due to Hawthorn effect, difficult to scale
		Laborious review and analysis of data	
		Hawthorne effect?	
Client satisfaction survey	Information on patient opinion and appreciation	Recall is a problem	Probably efficient, can be made responsive, scalable
	Information on out of pocket payments	Design and testing of the instrument is crucial	

The methods started to be used in the four countries of DRC, ROC, Kyrgyzstan, and Cambodia are: (i) Vignettes; (ii) Direct Observation and (iii) Client Satisfaction Surveys. Below, we describe how these methods are being applied in these four country contexts.

## METHODS – VIGNETTES IN PRACTICE

Vignettes are structured written or online case simulations that have been used in a wide variety of clinical [54-56] and non-clinical [57-59] settings. As described by Alexander and Becker (1978), vignettes, as originally conceived, are “short descriptions of a person or a social situation which contain precise references to what are thought to be the most important factors in the decision-making or judgment-making process of respondents” [60]. More recently new vignettes have been developed that are more sophisticated and require providers to care for every aspect of a patient, simulating an actual patient visit [54,61]. Clinical vignettes can simulate a range of medical conditions evaluating whether a physician or other provider has the skills/knowledge required to care for the patient. By standardizing patients, vignettes offer the possibility of examining different providers’ clinical interpretations and directly comparing their responses. Carefully constructed and validated vignettes have a better correlation with the “gold standard” – the mystery patient – than chart abstracts and they have been validated in developing or emerging countries, while they are a relatively inexpensive measure that can be applied at scale [14,20,54,61,62].

Across DRC and ROC 20 vignettes have been developed, customized to the local treatment guidelines, across 9 medical conditions; these are used at both health center and first-level referral hospitals. The DRC and ROC vignettes have been elaborated from vignettes used in the SDI surveys. Cambodia uses a total of 21 vignettes across 11 condition areas, some specific for the level of care (health center vs hospital level). Vignettes are administered by trained supervisors from the district health teams (for health centers) and trained peers and provincial health staff (for hospitals). The supervisor brings a “case” to the provider, who is randomly selected from the duty roster (hat method). Tablet-based software is used to enable feedback after the vignette session (in-service training component) and to automate upload to a cloud-based database for merging into the quality index. Feedback on performance is confidential and fed direct to the individuals concerned. Counter-verification of results is executed following a mixed systematic random and risk-based protocol, and is included in the existing counter-verification mechanism carried out by a third party. Fair play is encouraged through financial rewards, temporary exclusion from benefits for the offending supervisors and public benchmarking.

## RESULTS

### Direct observation in Cambodia and Kyrgyzstan

In Cambodia and Kyrgyzstan, direct observation, modified to a competency test, using the MamaNatalie and NeoNatalie tools, is also used. Evaluation of provider performance on these tools is performed by District and Provincial supervisors who have been trained and certified to carry out these assessments. MamaNatalie is used as a competency test for post-partum hemorrhage, one of the leading causes of maternal mortality, and the NeoNatalie is used for a competency test for neonatal resuscitation, which has been shown to decrease neonatal mortality by 30% [63]. Both competency tests have been quantified and weighted to score 100 points when 100% correctly executed. Both vignettes and competency tests are weighted a combined 30% in the hospital balanced score card (plus a 30% valuation on file reviews) and 60% in the health center balanced score card.

In **Table 4**, adjustments to these methods are described to make the methods dovetail with PBF approaches:

**Table 4:** Adjustments to vignettes, direct observations and client satisfaction surveys

Method	Adjustments	Countries
Vignette	Focus vignettes on top ten burden of disease	Cambodia; DRC; ROC; (Kyrgyzstan planned)
	Quantified and weighted	
	Use of trained district and provincial health supervisors	
	Third-party counter verification	
Direct observation	Modify to competency tests	Cambodia; Kyrgyzstan; (DRC and ROC planned)
	Quantified and weighted	
	Focus on major maternal and neonatal emergencies (post-partum hemorrhage and neonatal resuscitation)	
	At hospital level inclusion of the “WHO Surgical Safety Checklist” as a competency test (in Cambodia and Kyrgyzstan)	
	Use of trained district and provincial health supervisors	
	Third-party counter verification	
Patient satisfaction survey	Quantified and weighted	Kyrgyzstan; Cambodia; DRC and ROC
	Likert scales	
	Smart content of care tracers	
	Use of trained district and provincial health supervisors	
	Third-party counter verification	

DRC – Democratic Republic of the Congo, ROC – Republic of the Congo, WHO – World Health Organization

### Patient satisfaction surveys

Patient satisfaction surveys are being conducted in Kyrgyzstan. A systematic random sampling of the inpatient register of women who delivered at the hospital is used. Ten randomly selected patients from each of the 63 district hospitals are surveyed once per quarter. The patients are contacted through mobile phone by trained supervisors, and after obtaining consent are asked five questions. Four of the five questions have responses on a five-point Likert scale, while one final question is binary seeking to clarify whether the patient paid providers informally. If the last question is answered ‘yes’, then it voids the points for the entire interview. The ten interviews are weighted 10% of the total quarterly performance score for each hospital.

In Cambodia, DRC and ROC, the interview tool contains 15 questions, all quantified and on various scales, and weighted differentially. The tool contains content-of-care tracers such as questions related to taking blood pressure or temperature and whether the provider touched the belly of the patient or listened to the lungs. In Cambodia, patients are sampled from registers and called directly by the supervisors; while in DRC and ROC, larger community client satisfaction surveys are carried out by grassroots organizations, which validate the patient-provider contact (to deter phantom patients), in addition to running the patient experience questionnaire. In all three contexts, these surveys are weighted 10% of the total quality score.

**Box 1****,** **Box 2****,** **Box 3** and **Box 4** give short descriptions of the PBF programs in Kyrgyzstan, Cambodia and the two Congo’s.

Box 1 **The Kyrgyzstan Health Results-based financing Project**

Kyrgyzstan is a central Asian country with a 2016 population of 6 million. Its nominal 2015 GDP is US$1103 per capita, its 2016 human development index is 120/187. The Kyrgyz Health Results-based financing Project, a US$12M three-year project funded through a Health Results Innovation Trust fund grant (financed through Norway and the UK), was designed to impact on the relatively high maternal and early neonatal mortality in the Kyrgyz Republic. Kyrgyzstan has a relatively high maternal mortality ratio of 76/100 000 and a relatively high early neonatal mortality (Infant Mortality rate of 24/1000, 78% of these deaths occur within the first seven days after giving birth). These mortality figures are respectively 6 times and five times the OECD average. Whereas 99% of all women delivery in Hospitals, 64% of all mothers who die do this in Rayon (district) Hospitals, and this (and the early neonatal mortality) is due to poor quality hospital services.

Due to the systemic nature of the quality problems a balanced score card approach was developed targeting quality in Rayon hospitals. Causes of the quality problems in Rayon hospitals were multiple. Some of these causes were systemic and could not be solved as they were outside the control of hospitals (eg, rigid spending rules; relatively low salaries for human resources; brain drain). Other causes such as procedures, guidelines, stock management, quality assurance processes and knowledge and competency of providers were deemed to be solvable and under the influence of hospital management. A rigorous balanced score card (BSC) was developed with local practitioners, tested and piloted before scaling up in July 2014. This BSC is applied once per quarter to each hospital by a team of consultants, development partner technicians, and staff from the Mandatory Health Insurance Fund. Counter-verification of results is done by a team of consultants from central level. Due to the novel and experimental nature of the intervention a rigorous impact evaluation was conceived to study its effects [38]. An impact evaluation was designed with three arms: Group 1 Hospitals received a balanced score card with payment based on results, Group 2 Hospitals received a balanced score card with feedback but no payment and a third group where no intervention took place. The value of the performance bonus budget was about 15% of the annual hospital income, approximately US$1 per capita per year. Each of the groups had 21 hospitals. A baseline survey was carried out in 2013, and a follow-up survey has been done in the third quarter of 2017.

**Figure 2:**
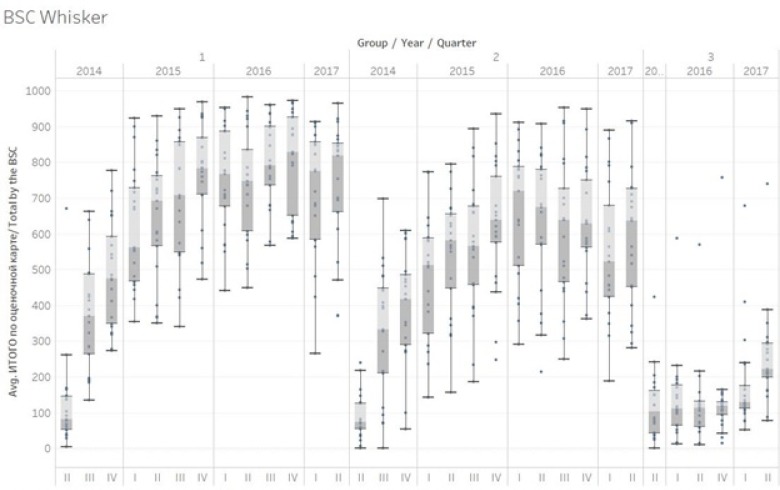
Kyrgyzstan 3-year balanced score card (BSC) results in the three impact evaluation groups.

Results over a two-year period showed impressive improvements in both intervention arms whereas the control arm remained the same. An average score of 9.8% was found for 63 Rayon hospitals in July 2014. Group 1 increased its performance over a period of two years from 9.3% to 79% (69.7 percentage point increase; double difference with Group 3 of 67.5 percentage points). Group 2 increased its performance from 8.6% to 60.8% (52.2 percentage point increase; double difference of 49.3%). These increases occurred notwithstanding changes to weightings after one year and introduction of a multitude of skill and competency based tests that put the performance bar progressively higher. Group 3 performance barely increased. Two rounds of balanced score card application was done to the Group 3 hospitals in the first half of 2016, and their performance remained low at 13.7%, representing a 2.2% increase from their July 2014 baseline of 11.5%. Kyrgyzstan RBF portal (http://rbf.med.kg/) (**Figure 2**).

Box 2 **The Republic of Congo (ROC) – PDSS2 program**

The Republic of Congo has a population of about 4.5 million people, a 2015 GDP of US$1851 per capita (dropped from US$3200 per capita per year in 2013). Its human development index is 136/187. A US$120 million five-year health system development project was conceived in 2013, based on a successful pilot experience in three departments [64], to extend PBF to cover 86% of the population. The project contains a component in which a health financing assessment is done with purpose to enhance fiscal space to move towards universal health coverage. This includes policy dialogue on human resources for health, the health information system and the pharmaceutical sector. Financing is based on US$100M Government funding, a US$10million IDA credit and a US$10 million grant from the HRITF. Budget for PBF is about US$7 per capita per year. Part of the intervention is an impact evaluation which divides the project in intervention and control areas [38]. The intervention districts cover 48% of the population. The control districts received decentralized facility financing without further interventions (this component is halted temporarily due to lack of Government financing caused by a drop in international oil prices and subsequent fall in Government revenues).

A basic package of health services of 20 services for health center and community level, are contracted to 195 public, private and quasi-public health centers. Three services from this package have a defined co-payment (the remainder of the services are free at the point of use) which is waived for indigents for which providers are paid a fee which covers all. A complementary package of 16 services is contracted to 17 district hospitals. Four services in this package have a defined co-payment (the remainder of the services are free at the point of use) which is waived for indigents for which hospitals are paid a fee which covers all. In the two cities of Brazzaville and Point Noire, over 75% of the contracts are with private for profit and quasi-public providers, which are offered a higher set of subsidies for their service packages.

Seven departmental health administrations and 21 district health administrations and four central ministry of health departments are under internal performance contracts. Four non-governmental organizations have been recruited to act as purchasing agencies. An external verification agent has also been recruited. A cloud based database with a public frontend with data upload through tablets and smart phones has been introduced (http://www.fbp-msp.org/#/).

Other interventions included a demand-generating project called the ‘Rainbow program’ in which color coded vouchers are used to identify those in need of key basic health services and to educate and persuade them to use services. In addition, in close collaboration with LISUNGI, the social protection program, 25% of the poorest households were identified and were issued ID cards to access services free of charge.

**Figure 3:**
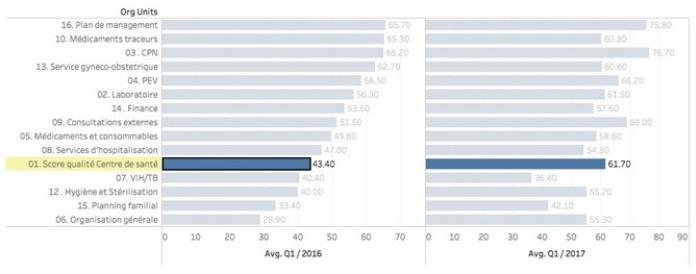
Republic of Congo: Quality Index in Health Centers increased from 43% to 62% over a one-year period.

Full program activities started in the first quarter of 2016, a significant increase in especially quality in health centers and hospitals has occurred, with a concomitant rise in key preventive services offered by contracted providers. In December 2017, new quality measures consisting of vignettes and MamaNatalie and NeoNatalie competency tests has been introduced. In addition, community client satisfaction surveys that measure and quantify client perceptions of serviced received have been introduced in the third quarter of 2017 (**Figure 3**).

Box 3 **The Cambodian H-EQIP program**

Cambodia is a Southeast Asian nation with a 2016 population of around 16 million. Cambodia has had a very fast GDP growth averaging 7.6% per year between 1994 and 2016. Its 2015 nominal GDP is US$1185 per capita. Its 2016 human development index is 143/187. Health system development after a period of experimentation, focused on financing health services for the poor through Health Equity Funds [27-30,65,66]. Despite dramatic improvements in maternal and child health, inequities persist across health outcomes by socioeconomic status, by geographical areas, and between urban and rural populations. The quality of health services in Cambodia is suboptimal. Preliminary findings from a recent World Bank study indicate that beneficiaries may be incurring high out-of-pocket payments due to the perceived poor quality of care in certain public facilities, even when they are covered by a health equity fund. In addition to some remaining gaps in infrastructure, Cambodia faces a major challenge with the skills and competencies of its health workforce and needs both pre-service and in-service training improvements and a renewed focus on competency-based training. In addition, the absence of a well-coordinated monitoring and evaluation mechanism and limited data quality have hampered the effective monitoring of health sector performance and evidence-based decision-making. The Cambodia Health Equity and Quality Improvement Project (H-EQIP) is a five year US$174 million project of which US$94 million is counterpart funding, US$30 million an IDA loan, and US$50 million is grant financing through a Multi-Donor Trust Fund (financed through Australia, Germany and South Korea). It was designed to continue funding and to scale up health equity funds nationwide, to bring in Government funding, and to strengthen quality of care in public facilities nationwide. From the project component related to the Service Delivery Grants, about US$1 per capita per year was available for performance based remuneration of quality.

**Figure 4:**
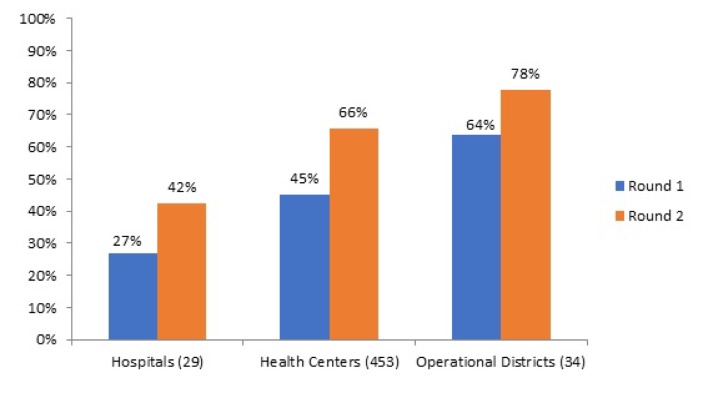
: Cambodia results from the first two rounds of ex-ante verification Health Center, Hospital and Health Administration performance (quarter 2, 2017; first wave of scaling up).

Tool development was done through a collaborative process. Use was made of existing vignettes that had been applied during a nationwide level 2 quality assessment. Balanced score cards were developed for health centers and three different type of hospitals (CPA-1; 2 and 3). At the health center level, structural quality was weighted 30% and consisted of financial management; health equity fund management and infection control, hygiene and medical waste disposal. Furthermore, knowledge and competency tests were weighted 60%, and client interviews 10%. At the hospital level the Kyrgyzstan balanced score card was used as a point of reference, and adjusted to local context. Weighing was 30% for structure, 60% for process (30% on file reviews and 30% on knowledge and competency tests) and 10% on client interviews. Tools developed were tested in pilot provinces and amended. Nationwide roll-out started in three waves in January 2017 and is planned to finish by June 2018. The scope of the quality intervention is nationwide; all public health facilities, all public hospitals and the entire district and provincial health administration are involved. Payment for results is based on the ex-ante verification by the health administration (also under a performance contract), whereas results will be counter-verified ex-post (after payment has taken place) by a third party. Tablet-based software will be developed, including a public dashboard with benchmarking of results. An impact evaluation is planned, using the phased introduction of the tools nationwide [38]. Ex-ante results for the first wave of verifications show a wide spread of quality performance in both health centers and hospitals, with significant upward potential (**Figure 4**).

Box 4 **The Democratic Republic of the Congo (DRC) - PDSS program**

The DRC is the second largest country by area and the third most populous country in Africa with an estimated population of over 70 million. Its nominal 2015 GDP is US$456 per capita. Its 2016 human development index is 176/187. Public resources spent to finance health amount to less than US$1 per capita per year and very little of this reaches the frontline health facilities with as consequence that the overall majority of public health workers do not receive a salary and that most cost at the point of use is financed by the population. From all resources spent on health, an estimated US$13 per capita per year, about 40% is spent by development partners, about 39% by households and less than 15% by the government. Out of pocket health expenditures account for over 90% of household expenses on health, and catastrophic health expenditures affect more than 10% of households [67]. The Health System Strengthening Program is a five-year US$400 million program financed through an IDA grant of US$210 million, an IDA loan of US$130 million, about US US$50 million grants through trust-fund sources (GFF; HRITF and USAID) and a US$10 million grant from the Global Fund. The overall financing goes towards a PBF program covering 156 health zones across 11 provinces covering an estimated 30% of the population at a cost of US$3.60 per capita per year. In the PBF program, the largest of its kind in the world, both quantity and quality of health services are purchased through a set of 22 services at the health center and community level, and a set of 24 services at the first referral hospital level, both quality adjusted through a quality checklist (separate indices for the two levels). Service packages are focused on maternal and child health preventive and promotive activities and infectious diseases such as tuberculosis, malaria and HIV, extensive community based activities in addition to access to curative care. Health Districts and Provincial Health Departments are under performance contracts to strengthen their tasks which include quarterly quality assessments in health centers and hospitals. New purchasing agencies have been set up as ‘public utilities’; four main public utilities with ten satellite offices have been put in place. Staff have been recruited through a transparent merit-based procedure with participation of development partners (UNICEF; Cordaid; SANRU) and trained rigorously. An independent counter-verification agent has been recruited. Roll out was completed by the end of 2016, and the system started in January 2017, with over 3150 contracts. Cloud based software with a public frontend and the use of tablets and smart phones for data collection and upload to the database have been implemented (http://front.fbp-rdc.org/#/republique-democratique-du-congo/g/pL5A7C1at1M).

**Figure 5:**
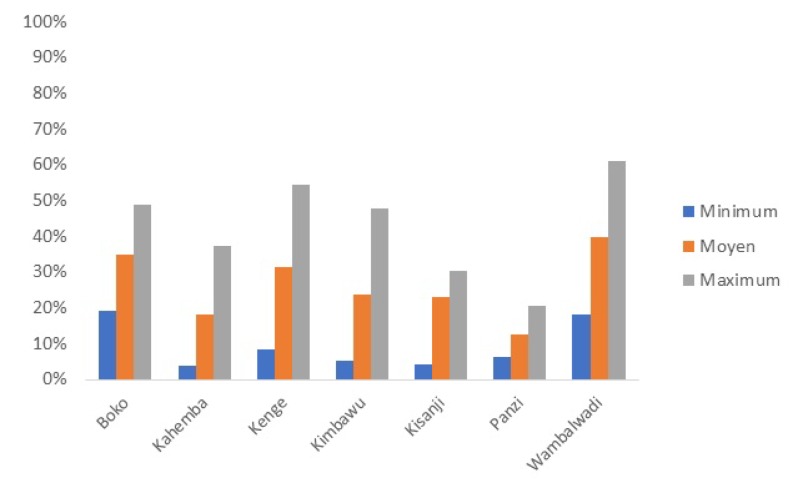
Democratic Republic of Congo. Lowest, Average and Maximum Quality Index Scores at Baseline for health centers in the six health districts of Kwango Province, Q3, 2016.

Due to the vast expanse of DRC, and because the project covers some of the most distant areas in DRC such as Equator and Bandundu, a system of geographic equity bonuses has been put in place. Subsidies for service packages depend on travel time to the center of the district and the provincial capital and are a proxy for remoteness. There are nine sets of health center subsidies with those in the most remote regions 80% higher than in those in the lowest category. Similarly, health districts and district hospitals are allocated in five equity categories with the ones furthest away from the province center receiving a 25% higher (districts) and 40% higher (hospitals) performance-based subsidy. A rigorous impact evaluation is included it its design aimed at researching the impact of the PBF scheme including innovative interventions to boost quality of care through the addition of vignettes and competency tests to the quality index [38]. Baselines for quality are very low, for instance in Kwango province, the average quality index for health centers is 26% (Min 12.5%; Max 40.4) (**Figure 5**).

## DISCUSSION

As PBF develops and builds towards UHC, there is an opportunity for new methods to strengthen the quality of service delivery. From field observations and routine data collection it appears that quick improvements in key quality measures can be obtained by integrating several state-of-the-art quality methods into the PBF armamentarium. In four country examples, PBF quality of care improvement programs are collecting evidence in rigorous impact evaluations. Ongoing experience from the four country examples show that the methods proposed are feasible, and appropriate. Early results from the impact evaluation of the Kyrgyzstan results-based financing approach show strong impact of the balanced score card with its robust weighting for content of care measures such as knowledge and competency testing, and client interviews.

However, experiences from the novel methods used until now have revealed a set of theoretical and actual challenges, which can be classified into micro level challenges (the methods, tools and reforms) and macro-level ones (political economy).

### Micro level

At the micro level, there is the conceptual challenge of the *know-do gap*, with *design and implementation of these novel quality tools* in general, and *design and implementation of PBF approaches* specifically. The know-do gap [8,38], is an observed gap between what providers know and what they do in practice with that knowledge [38. This know-do gap is a problem in knowledge-based tests. For example, providers with a high score on the vignettes and competency tests might systematically skimp on actual care. We know very little about this phenomenon in PBF contexts. However, a rigorous impact evaluation in Rwanda showed that under PBF knowledgeable providers did more of what they knew [39,68]. A study in China on tuberculosis diagnoses found that providers knew much more as tested through vignettes, than they did as tested through mystery patients. Although this study was not in a PBF context, incentives linked to prescribing antibiotics and other drugs might have had an influence on the discrepancies [61,62]. A well-designed randomized controlled study in the Philippines showed that doctors who did better on a comprehensive set of vignettes designed so that they were difficult to game, improved their care and these improvements were reflected in better health outcomes [14].

There are a few possible ways to mitigate this challenge of the know-do gap with vignettes. First, direct feedback to the providers on their vignette results (individual learning element), and provider effort is helpful for learning. This is enhanced by structured formative supervision, a key component of PBF approaches. Second, the change in provider payment mechanism (moving to a case-based payment for curative care instead of an itemized billing) aligns incentives to prescribe drugs or initiate treatment more rationally, ie, adhere to protocols (taught through the vignettes). Third, perceived enhanced quality by patients (observed structural elements; more time spent in consultation) leads to a positive virtuous cycle [69]. Fourth, observation with positive feedback, and benchmarking of health facility performance (individual health worker performance is not made public) are all strong enablers for enhancing performance. And finally, linking health facility performance payments to these novel quality measures are intuitively appealing as they counter some of the negative effects of routine vignette applications [61,70,71]. Ultimately, the know-do gap must be measured not by gaps in performance but by whether serial measurement and feedback leads to improvement as measured by health outcomes.

While scalable in practice, the design and implementation of these novel quality tools holds another set of challenges [24,26,47,72]. Field observations from Kyrgyzstan and Cambodia have taught us that continued high quality mentoring and support are necessary pre-conditions to measurement implementation. Investments need to be made in creating capacity of a group of national trainers who will be able to continue implementing and developing these measurement approaches. With training and measurement capacity, a necessary ‘bottom-up’ design and approach can be used to strengthen local acceptance, gather inputs, and ensure adaptation of quality measurement tools. A period of instrument validation may be required to ensure validity within the local context [18]. A further implementation challenge for these approaches is the use of local administrative structures for the assessments. Whereas engaging the local health administration is a powerful strategy for strengthening local health systems, using PBF approaches necessitates very good counter-verification measures to make all adhere to the new rules of the game.

### Macro level

At the macro level, there are political challenges. Countries that do not invest in PBF or do not create a line item for PBF and quality measurements in the Ministry of Finance budget, pose a sustainability risk to these reforms. Successful reforms such as in Rwanda and Burundi were brought about by Governments that planned for a budget to finance PBF. Leadership changes pose further challenges to sustainability. A new team might have different priorities and could derail these reforms. Also, development partner buy-in is crucial in many contexts. Other macro challenges to PBF implementation come from those that stand to lose out due to these reforms. For instance, decentralizing drug procurement and decentralizing budgets to health facilities all lead to lesser income for those that previously used to manage these resources at higher levels.

Finally, in many countries weak clinical training is the root cause of poor provider performance. Implementing novel quality measurement and enhancement methods will certainly be beneficial, however, it will not be the lone solution. Deeper reforms of the pre-service medical training curricula will be required. For example, once the habit of measurement and feedback has been established, these could also be applied to the pre-service curricula.

## CONCLUSIONS

Implementing Universal Health Coverage is a long and arduous road especially for lower- and middle-income countries, which are under pressure to invest in health next to a legion of other priorities. For countries that have Performance-based financing programs, however, there is a highly leverageable opportunity to enhance UHC. There is great potential to impact the quality of care using newer measurement methods. Although the methods described in this article are novel in the context of PBF and LMIC, they are being tested and there is hope that with relatively minor adjustments provider knowledge and competency can be rapidly improved.

## 

**Table 5:** Average quality index for health centers in three provinces, 30 health districts, Democratic Republic of the Congo

			2016	2017				2018
**Province**	**Health zone**	**Structures**	**3**	**1**	**2**	**3**	**4**	**1**
Kwango	Boko	CS	34.4	52.1	66.9	67.6	45.0	39.7
	Kahemba	CS	16.5	34.8	55.3	63.7	34.4	34.1
	Kenge	CS	29.5	54.4	70.0	74.9	40.9	38.4
	Kimbao	CS	25.7	52.4	70.3	74.6	29.5	29.1
	Kisandji	CS	22.9	47.0	65.8	64.1	31.1	31.4
	Panzi	CS	11.5	28.7	59.3	68.3	35.1	33.2
	Wamba Lwadi	CS	39.2	50.7	53.7	60.9	32.2	33.3
Kwilu	Bandundu	CS	32.3	55.8	70.8	76.0	40.6	82.6
	Djuma	CS	14.6	42.7	56.8	57.3	34.4	72.3
	Gungu	CS	12.0	29.4	44.6	48.2	29.2	60.2
	Kikwit Nord	CS	25.1	48.7	66.6	78.5	41.0	84.5
	Kimputu	CS	26.6	53.3	52.9	57.6	31.2	60.2
	Kingandu	CS	13.9	30.0	48.1	59.9	31.8	68.3
	Moanza	CS	11.4	33.5	49.9	51.3	32.6	61.7
	Mosango	CS	38.3	55.9	66.1	76.7	40.0	77.4
	Mukedi	CS	28.9	26.7	36.5	55.7	31.5	65.7
	Mungindu	CS	11.8	51.4	73.1	53.2	30.5	64.4
	Pay Kongila	CS	29.2	48.2	63.1	76.0	36.6	72.0
	Sia	CS	20.9	42.0	48.9	51.2	28.1	53.6
	Vanga	CS	15.6	54.2	64.8	73.4	38.4	74.8
Maindombe	Bokoro	CS	24.8	44.5	54.2	63.5	34.4	70.2
	Bolobo	CS	15.1	31.3	47.1	56.8	30.9	64.8
	Inongo	CS	14.6	41.1	61.1	64.6	32.3	67.9
	Kwamouth	CS	15.5	32.9	57.4	62.0	26.9	62.2
	md Kiri	CS	8.2	26.7	50.3	66.6	33.0	69.0
	Mushie	CS	25.5	44.3	60.7	62.8	67.9	68.0
	Nioki	CS	18.2	59.5	63.4	73.0	35.3	70.8
	Ntandembelo	CS	6.9	18.9	37.0	59.1	32.9	61.2
	Pendjwa	CS	26.9	37.4	66.4	68.7	34.8	68.6
	Yumbi	CS	18.1	45.1	60.0	69.1	31.6	62.5

CS – Centre de Sante (health center)
